# The effect of mesenchymal stem cell conditioned medium incorporated within chitosan nanostructure in clearance of common gastroenteritis bacteria in-vitro and in-vivo

**DOI:** 10.1038/s41598-024-64465-y

**Published:** 2024-06-20

**Authors:** Sareh Bagheri-Josheghani, Mahmood Saffari, Tooba Radaei, Hamed Mirzaei, Somaye Rashki, Zahra Sadat Fatemi-Nasab, Elahe Derakhshan-nezhad, Bita Bakhshi

**Affiliations:** 1 Infectious Diseases Research Center, Kashan University of Medical Sciences, Kashan, Iran; 2 Department of Microbiology and Immunology, Faculty of Medicine, Kashan University of Medical Sciences, Kashan, Iran; 3 Department of Medical Bacteriology, Faculty of Medical Sciences, Tarbiat Modares University, Tehran, IR Iran; 4 Research Center for Biochemistry and Nutrition in Metabolic Diseases, Institute for Basic Sciences, Kashan University of Medical Sciences, Kashan, Iran; 5 Student Research Committee, Kashan University of Medical Sciences, Kashan, Iran; 6 Department of Clinical Microbiology, Iranshahr University of Medical Sciences, Iranshahr, Iran; 7 Faculty of Medicine, Mashhad University of Medical Sciences, Mashhad, Iran

**Keywords:** Antibacterial, Antibiofilm, Mesenchymal stem cells, Chitosan, Gastroenteritis, Microbiology, Stem cells

## Abstract

Gastroenteritis infection is a major public health concern worldwide, especially in developing countries due to the high annual mortality rate. The antimicrobial and antibiofilm activity of human mesenchymal stem cell-derived conditioned medium (hMSCsCM) encapsulated in chitosan nanoparticles (ChNPs) was studied in vitro and in vivo against common gastroenteritis bacteria. The synthesized ChNPs were characterized using Zeta potential, scanning electron microscopy (SEM), and dynamic light scattering (DLS) techniques. HMSC-derived conditioned medium incorporated into chitosan NPs (hMSCsCM-ChNPs) composite was fabricated by chitosan nanoparticles loaded with BM-MSCs (positive for CD73 and CD44 markers). The antimicrobial and antibiofilm activity of composite was investigated against four common gastroenteritis bacteria (*Campylobacter jejuni* ATCC29428, *Salmonella enteritidis* ATCC13076, *Shigella dysenteriae* PTCC1188, and *E. coli* ATCC25922) in-vitro and in-vivo. Majority of ChNPs (96%) had an average particle size of 329 nm with zeta potential 7.08 mV. The SEM images confirmed the synthesis of spherical shape for ChNPs and a near-spherical shape for hMSCsCM-ChNPs. Entrapment efficiency of hMSCsCM-ChNPs was 75%. Kinetic profiling revealed that the release rate of mesenchymal stem cells was reduced following the pH reduction. The antibacterial activity of hMSCsCM-ChNPs was significantly greater than that of hMSCsCM and ChNPs at dilutions of 1:2 to 1:8 (P < 0.05) against four common gastroenteritis bacteria. The number of bacteria present decreased more significantly in the group of mice treated with the hMSCsCM-ChNPs composite than in the groups treated with hMSCsCM and ChNPs. The antibacterial activity of hMSCsCM against common gastroenteritis bacteria in an in vivo assay decreased from > 10^6^ CFU/ml to approximately (102 to 10) after 72 h. Both in vitro and in vivo assays demonstrated the antimicrobial and antibiofilm activities of ChNPs at a concentration of 0.1% and hMSCsCM at a concentration of 1000 μg/ml to be inferior to that of hMSCsCM-ChNPs (1000 μg/ml + 0.1%) composite. These results indicated the existence of a synergistic effect between ChNPs and hMSCsCM. The designed composite exhibited notable antibiofilm and antibacterial activities, demonstrating optimal release in simulated intestinal lumen conditions. The utilization of this composite is proposed as a novel treatment approach to combat gastroenteritis bacteria in the context of more challenging infections.

## Introduction

Gastroenteritis is a major public health concern worldwide, especially in developing countries. The disease causes about two million deaths per year in children under 5 years of age in developing countries alone. Diarrhea syndrome is rarely fatal, except in the elderly and immunocompromised individuals; however, it remains as an important cause of morbidity^1^. Antibiotic therapy represents the optimal treatment for infections caused by bacteria. However, the necessity for developing novel non-antibiotic agents capable of combating bacterial infections is becoming increasingly challenging, particularly in the context of infections that are untreatable or difficult to treat with conventional antimicrobials. In the present era, researchers are engaged in the search for novel non-antibiotic therapeutic approaches to address challenging infections^2^.

In the previous decades, nanotechnology has exhibited great potential in the fields of biomedicine owing to its special nanoscale chemical-physical features, biocompatibility, and biosafety. Todays, nanomaterials are employed as effective tools for the controlled delivery and release of drugs with minimal side effects^3^. As a valuable biopolymer, chitosan (Ch) has excellent physicochemical properties. In recent years, chitosan and its derivatives have been extensively investigated for their antimicrobial properties and have shown potent antimicrobial and antibiofilm activities against fungi as well as Gram-negative and Gram-positive pathogens.

Furthermore, chitosan nanoparticles (ChNPs) increase intestinal permeability due to the presence of cellular receptors on M and dendritic cells; in addition, chitosan nanoparticles directly mediate bacterial killing by improving humoral and cellular immunity. Recently, it has been shown that chemical alteration and conjunction of chitosan with existing antimicrobial substances improves its antimicrobial effects^4,5^.

Antibacterial activities of mesenchymal stem cells (MSCs) extracted from different adult tissues have been described^6,7^. MSCs secrete many bioactive compounds with different anti-inflammatory, anti-apoptotic, chemotactic, and antimicrobial effects. Also, MSCs with anti-inflammatory properties and low-toxicity could downregulate the expression of major matrix components. MSCs could secrete antimicrobial peptides (AMPs) and improve the antimicrobial activities of certain antimicrobials. Several investigations have shown the high potential of MSCs in removing bacteria from an infected area in several bacterial infections, such as cystic fibrosis (CF) infection, sepsis, as well as acute respiratory distress syndrome (ARDS)^8,9^. Secreted AMPs of MSCs could damage bacterial membrane integrity. Also, it could cause damage and depolarization of bacterial cell membranes. MSCs and MSCs CM have been shown to inhibit the growth of bacteria^9,10^. For example, MSCs have been used as an adjunct treatment for *E*.*coli* infections^11,12^. In a study, soluble substances produced by MSCs showed levels of spontaneous direct bactericidal activity in-vitro and inhibited *S. aureus* biofilm formation. Therefore, MSC therapy is considered as a new therapeutic approach that could be used to treat bacterial infections^7^. Although the effects of MSCs and chitosan have been extensively evaluated, further investigations are required to elucidate the antibiofilm and antibacterial properties of MSCs and chitosan, with the aim of achieving new strategic progress in the treatment of infectious diseases such as gastroenteritis infections. In this research, the antibiofilm and antibacterial activities of hMSCsCM incorporated within ChNPs against common gastroenteritis bacteria were evaluated in-vitro and in mice models.

## Materials and methods

### Ethical approval

Ethical clearance was obtained from the Ethics Committee of Kashan University of Medical Sciences (IR.KAUMS.MEDNT.REC.1400.194).

### Bacterial strains

In the present study, *Campylobacter jejuni* ATCC29428, *Salmonella enteritidis* ATCC13076, *Shigella dysenteriae* PTCC1188, and *E. coli* ATCC25922 isolates were prepared from our collection in Department of Bacteriology, Tarbiat Modares University (Tehran, Iran). The prepared isolates were cultured in 1 mL of BHI (brain heart infusion) broth (Merck, Germany) at 37 °C. The absorbance of bacterial suspension was measured at 540 nm, until reaching the log phase (optical density (OD): 1:0). Finally, the bacterial samples were corrected to the favorable concentration.

### Preparation of human bone marrow-isolated mesenchymal stem cells (hBM-MSC) conditioned medium

Both Caco-2 cells and bone marrow-isolated mesenchymal stem cells (BM-MSCs) used in this study were obtained from the Iranian Biological Resource Center. BM-MSCs were differentiated into adipocyte and osteoblast lineages (two mesenchymal lineages) using an immunohistochemistry (IHC) assay and CD73, CD45, CD44, and CD34 markers confirmed using flow cytometry as previously described by Dominici et al.^13^.

The conditioned medium (CM) of hBM-MSCs were isolated and expanded, as reported previously with some modifications. The conditioned medium (CM) of hBM-MSCs was prepared from 1 × 10^6^ BM-derived mesenchymal stem cells. After reaching 90% confluency, BM-MSCs were subjected to the 5 ml of serum-free DMEM cell culture medium (Sigma, Aldrich) cultivation for 48 h. Then the culture medium was subjected to a short 10-min centrifugation step at 1800 *g* and 25 °C to remove cell pellets. In total, 0.22 μm filter-sterilized supernatant of BM-derived mesenchymal stem cells was collected and chosen as hBM-MSC-CM and maintained at – 80 °C up to the use. Caco-2 cells conditioned medium were used as control.

### Preparation of chitosan nanoparticles (ChNPs) and hMSCsCM-ChNPs formulation

We prepared the ChNPs with a concentration of 0.1% by the ionic gelation method via the interaction with sodium tripolyphosphate (TPP) according to the method described in literature with some modifications^14^. Briefly, 5 mL of low molecular weight Chitosan (Ch) solution (Sigma-Aldrich, USA) with a 50–200 kDa molecular weight was dissolved in 1% v/v acetic acid (Merck, Germany). Then, sodium tripolyphosphate (TPP) was mixed with phosphate-buffered saline (PBS) for chitosan nanoparticle synthesis and MSCs CM used for MSCs CM-CS NPs composite synthesis (ratio: 1:3) to prepare TPP solution. Finally, aqueous solution of TPP 2 ml was added into 5 ml Ch solution (ratio: 2:5) via a syringe needle under stirring at RT. Particle size, polydispersity index (PDI) of ChNPs dispersed in water were measured using dynamic light scattering analyses (DLS) in room temperature by a Zetasizer Nano Series (Zetasizer Nano ZS, Malvern, Worcestershire, UK). In order to study the morphology of ChNPs and MSCs CM-CS NPs composite were determined by scanning electron microscopy (FESEM, Tescan Mira3). The present research was conducted on three structures as follows: MSC-derived conditioned medium (hMSCsCM; 1000 μg/ml), chitosan NPs (ChNPs; 0.1%), and MSC-derived conditioned medium incorporated into chitosan NPs (hMSCsCM-ChNPs; 1000 μg/ml + 0.1%) Table 1 shows the formulation of ChNPs and hMSCsCM-ChNPs in the current research.

**Table 1:** Formulation of ChNPs and hMSCsCM-ChNPs.

Composite	TPP concentration	CS concentration	MSCs CM concentration	TPP solution ratio
CS NPs	500 ml, 1500ml PBS	0.1%	–	TPP: MSC CM 1:3
MSCs CM-CS NPs	500 ml, 1500ml MSCs CM	0.1%	1000μg /ml	TPP: PBS 1:3

### Evaluation of protein loading and in-vitro Release pattern

The amount of protein encapsulated in hMSCsCM-ChNPs was measured at 565 nm by the BCA (bicinchoninic acid) assay kit. The protein release pattern was evaluated in*-*vitro using a model as reported by Deng, et al.^15,16^ with some modifications at pH = 1.2 and 7.4 at 37 °C during 72 h using the BCA protein quantification kit. Protein release pattern was measured at different time intervals. Accordingly, protein-containing ChNPs were dissolved in 1 mL of PBS (10 mM) at pH = 1.2 and 7.4 at 37 °C. After 1, 2, 3, 4, 24, 48, and 72 h, 0.5 mL of the supernatant was picked up and subjected to centrifugation at 5000 × *g* for 20 min; then fresh PBS with an equal volume (0.5 mL) was added to the supernatant. Finally, the BCA assay kit was used to measure the concentration of protein in the supernatant. PBS solution was regarded as a blank sample. All tests in the release assay were repeated twice. The amount of protein released in the supernatant was measured using the BCA assay kit. Protein release was determined by dissolving protein-loaded nanoparticles in 1mL of 10mM phosphate-buffered saline (PBS) at pH = 7.4 and 1.2 at 37 °C. hMSCsCM-ChNPs solution was replaced with PBS at 1, 2, 3, 4, 24, 48, and 72 h time intervals.

### Determination of efficacy of hMSCsCM-ChNPs composite in inhibition of bacterial growth by Broth Microdilution method

The antibacterial effects of the prepared composite (hMSCsCM-ChNPs) and its components (hMSCsCM and ChNPs) against common gastroenteritis bacteria (*S. dysenteriae* PTCC1188, *S. enterica* ATCC13076, *C. jejuni* ATCC29428, and *E. coli* ATCC25922) was assessed using broth microdilution as described previously with some modifications and colony counting methods according to Clinical and Laboratory Standards Institute (CLSI) guidelines. In this method, 100 μL of LB (Luria–Bertani) broth medium was added to all wells. The first well in each row was filled with 100 µL of the highest concentration of of hMSCsCM-CS NPs, ChNPs, and hMSCsCM was transferred to the second well, and this process was continued to the final well. As result, this component was serially diluted (1:2 to 1:64). Finally, microplates were inoculated 0.01 ml of bacterial suspensions (5 × 10^5^ CFU/mL) of each bacteria were added to the wells, and the wells were subjected to incubation at 37 °C. Following 24 h incubation, the optical density was read at 620 nm and CFU of bacterial cells was calculated. Wells containing of bacteria without antimicrobials as well as 100 μl Caco2 cells supernatant (instead of MSCs supernatant) were run in parallel in each experiment and regarded as bacterial growth control^8^.

### Determination of efficacy of hMSCsCM-ChNPs composite in inhibition of bacterial biofilm formation by Crystal Violet method

In the current research, the antibiofilm activities of the prepared composite (hMSCsCM-ChNPs) and its components (ChNPs and hMSCsCM) against the following common gastroenteritis bacteria were assessed using the crystal violet staining method in 96-well microplates as previously explained^17^: *S. dysenteriae* PTCC1188, *S. enterica* ATCC13076, *C. jejuni *ATCC29428, and *E. coli* ATCC25922. In brief, 100 μL of LB broth + 2% w/v sucrose was transferred into 96-well microplates and serially diluted (1:2 to 1:64), then 0.1 mL of the new composite (hMSCsCM-ChNPs) and its components (ChNPs and hMSCsCM) were added to the wells. Then 10 μL of bacterial cell suspensions (10^8^ CFU/mL) of the above-mentioned gastroenteritis bacteria were added to the wells and incubated at 37 °C for 24 h. Adherent cells were then washed using distilled water and subjected to staining with 1% crystal violet. Ethanol (95%) was used for 40 min to de-stain the wells. The optical density (OD) of crystal violet associated with biofilm formation was defined at 570 nm. The supernatant of Caco-2 cells as well as bacteria without any antimicrobial were used as controls. This test was performed in triplicate.

### In- vivo antibacterial activity in infected mice

In- vivo antibacterial activities of the new composite (hMSCsCM-ChNPs) and its components (ChNPs and hMSCsCM) against common gastroenteritis bacteria were evaluated in infected mice. All animal studies were carried out in accordance with relevant guidelines and regulations and ARRIVE guidelines. This experiment was performed on 6-week-old male BALB/c mice obtained from Razi Institute (Karaj, Iran). Animal tests were conducted taking into account the related regulations and guidelines of the Institute of Laboratory Animal Resources. In this experiment, the mice were randomly divided into six groups of six and kept in distinct cages under normal temperature and light conditions. Mice models of bacterial infections were prepared by gavage of 100 μL of bacterial cells of common gastroenteritis bacteria (*S. dysenteriae* PTCC1188, *S enterica* ATCC13076, *C. jejuni *ATCC29428*,* and *E. coli* ATCC25922). PBS-administered mice were regarded as negative control. After 24 h, the mice were treated with 100 μL of the new composite hMSCsCM-ChNPs, ChNPs, and hMSCsCM separately once a day for 3 days. Fecal samples of mice were collected at 0, 24, 48, and 72 h before and after treatment and added to 1 mL of PBS. The samples were diluted in PBS at a ratio of 1:10 (w/v), centrifuged at 3000 r/min, and homogenized. Supernatants were cultured on specific culture media (*E. coli*: EMB, *C. jejuni*: Brucella agar, *Salmonella* and *S.* *dysenteriae*: SS agar) and incubated at 37 °C for 24 h. Finally, the number of bacterial colony-forming units on the plates were counted. Table 2 shows the groups of mice treated in the current research^18^.

**Table 2:** The groups of mice treated in the current research.

Group	Variable
Group 1	hMSCsCM (1000 μg/ml)
Group 2	ChNPs (0.1%)
Group 3	hMSCsCM-ChNP (1000μg/ml + 0.1%)
Group 5	Caco-2 cells (positive control)
Group 6	PBS (negative control)

### Statistical analysis

Statistical data were analyzed by GraphPad Prism software, Version 8 (GraphPad Software, Inc., 160 USA). Significant differences between groups were analyzed by ANOVA test. All obtained results were reported as mean ± standard deviation (SD). *P* values of less than 0.1 were regarded as statistically significant. All experiments were repeated three times.

### Ethics approval and consent to participate

The present research was approved by the Ethics Committee of Kashan University of Medical Sciences (IR.KAUMS.MEDNT.REC.1400.194).

## Results

### Characterization of MSCs

To characterize bone marrow-derived mesenchymal stem cells, the cells were differentiated into adipocyte and osteoblast lineages (two mesenchymal lineages) using an immunohistochemistry (IHC) testing method. Bone marrow-derived MSCs were categorized based on flow cytometric analysis of CD73, CD44, CD45, and CD34 markers. According to the results, MSCs were positive for CD73 and CD44 but negative for CD45 and CD34 as standard hematopoietic cell-surface markers^8^.

### Characterization of nanoparticles

**Figure 1:**
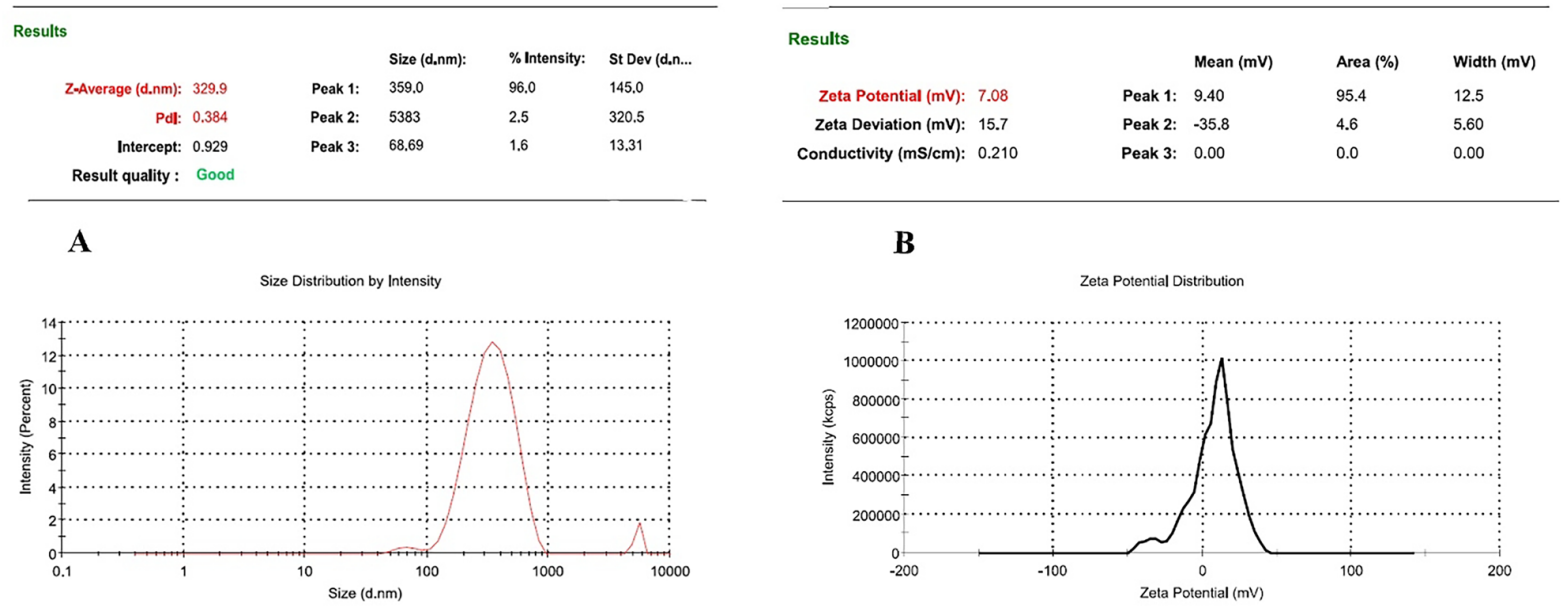
(**A**) DLS analysis results revealed an average particle size of 329 nm and (**B**) a zeta potential of 7.08 mV for the synthesized ChNPs.

**Figure 2:**
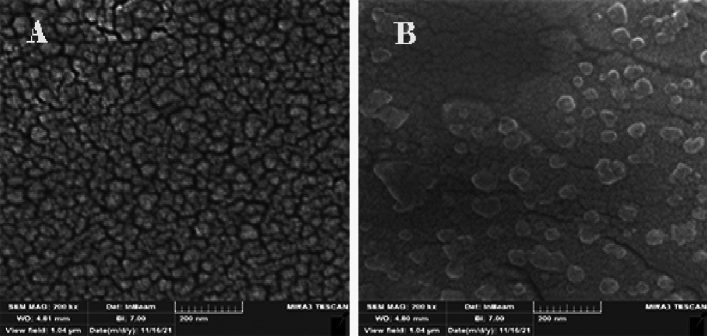
Morphology of ChNPs and the prepared composite (hMSCsCM-ChNPs): (**A**) SEM imaging of ChNPs, (**B**) SEM imaging of the prepared composite (hMSCsCM-ChNPs).

Dynamic light scattering (DLS) technique was used to determine the zeta potential, particle size, and polydispersity index (PDI) of ChNPs by employing a Zetasizer Nano Series instrument (Zetasizer Nano ZS, Malvern, Worcestershire, UK). The size distribution and electrical charge of ChNPs were ~ 329 nm and 7.08 mV, respectively (Fig. 1A and B). The SEM analysis results revealed a spherical shape for ChNPs (Fig. 2A) and a near-spherical shape with a smooth and irregular outer surface for ChNPs crosslinked with hMSCsCM (hMSCsCM-ChNPs) (Fig. 2B).

### Entrapment efficiency (EE %) and in- vitro release assay

**Figure 3:**
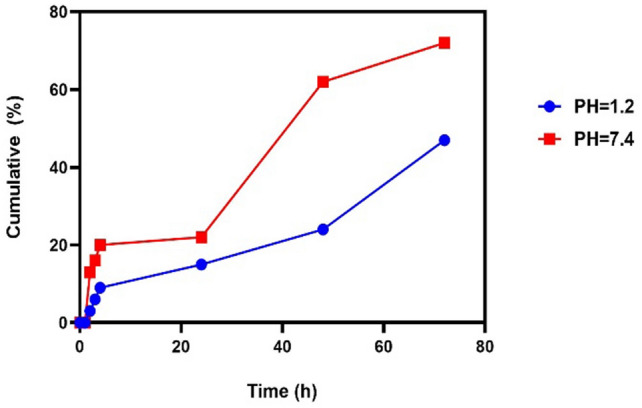
In-vitro release rate of hMSCsCM from hMSCsCM-ChNPs at two different pH values (pH = 7.4 and pH = 1.2).

The entrapment efficiency (EE) of the prepared composite (hMSCsCM-ChNPs) was 75%, demonstrating the entrapment of significant amounts of hMSCsCM in ChNPs. The protein (hMSCsCM) release profile from the prepared composite (hMSCsCM-ChNPs) was evaluated using phosphate-buffered saline during a period of 72 h at different pH values (pH = 1.2 and 7.4), the obtained results are shown in Fig. 3. The release rate of protein from the composite decreased with decreasing pH. The protein (hMSCsCM) release rate from the composite (hMSCsCM-ChNPs) at pH 7.4 was 0, 13, 62, and 72% after 1, 2, 48, and 72 h, respectively. Also, the protein (hMSCsCM) release rate from the composite (hMSCsCM-ChNPs) at pH = 1.2 was 0, 3, 24, and 47% after 1, 2, 48, and 72 h, respectively (Fig. 3).

### Antimicrobial efficacy evaluation by Broth Microdilution method

**Figure 4:**
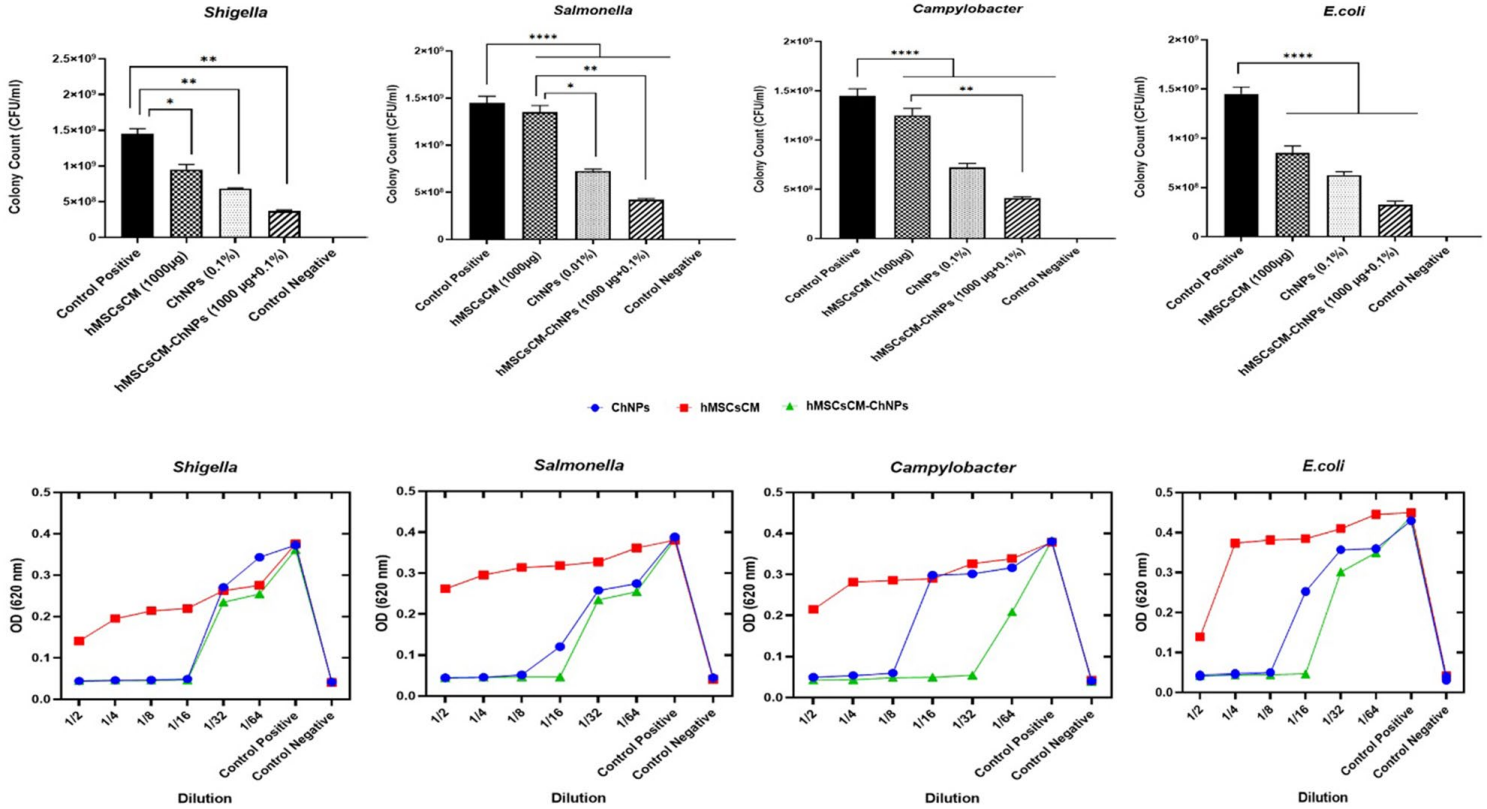
Antibacterial activities of hMSCsCM-ChNPs, ChNPs, and hMSCsCM against common gastroenteritis bacteria. The growth of bacteria was evaluated by counting CFUs. Bars represent the average results of triplicate experiments ± SD. The obtained results were analyzed by ANOVA test. P < 0.05: the mean difference was significant, *p-value < 0.05, **p-value < 0.01, ***p-value < 0.001, ****p-value < 0.0001, and *ns* no significant.

The antibacterial activities of hMSCsCM, ChNPs, and the composite (hMSCsCM-ChNPs) against common gastroenteritis bacteria (*S. dysenteriae* PTCC1188*, S. enterica* ATCC13076, *C. jejuni *ATCC29428, and *E*. *coli* ATCC25922) were assessed by broth microdilution assay. The new construct (hMSCsCM-ChNPs) exhibited a higher inhibitory activity against the growth of bacteria compared to each of its components. The antibacterial activity of hMSCsCM-ChNPs compared to hMSCsCM was significant against *C*. *jejuni* ATCC 29428 and *S. enterica* ATCC 13076. According to the findings, the antimicrobial activities of ChNPs at a concentration of 0.1% and hMSCsCM at a concentration of 1000 μg/ml (0.1 mL) were lower than that of hMSCsCM-ChNPs prepared with the same concentrations. Indeed, ChNPs and hMSCsCM separately were able to inhibit the growth of bacteria at concentrations higher than 0.1% and 1000 μg/ml, respectively. The antibacterial activity of hMSCsCM-ChNPs at 1:2 to 1:8 dilutions was significant than those at dilutions of 1:32 to 1:64 (P < 0.05) against common gastroenteritis bacteria (*S. dysenteriae* PTCC1188, *S. enterica* ATCC13076, *C. jejuni* ATCC29428, and *E. coli* ATCC25922). These findings highlight the synergistic effect of hMSCsCM and ChNPs. The antibacterial activities of ChNPs, hMSCsCM, and hMSCsCM-ChNPs (1:2 to 1:64) against common gastroenteritis bacteria are shown in Fig. 4.

### Antibiofilm assay by microtiter plate-crystal violet method

**Figure 5:**
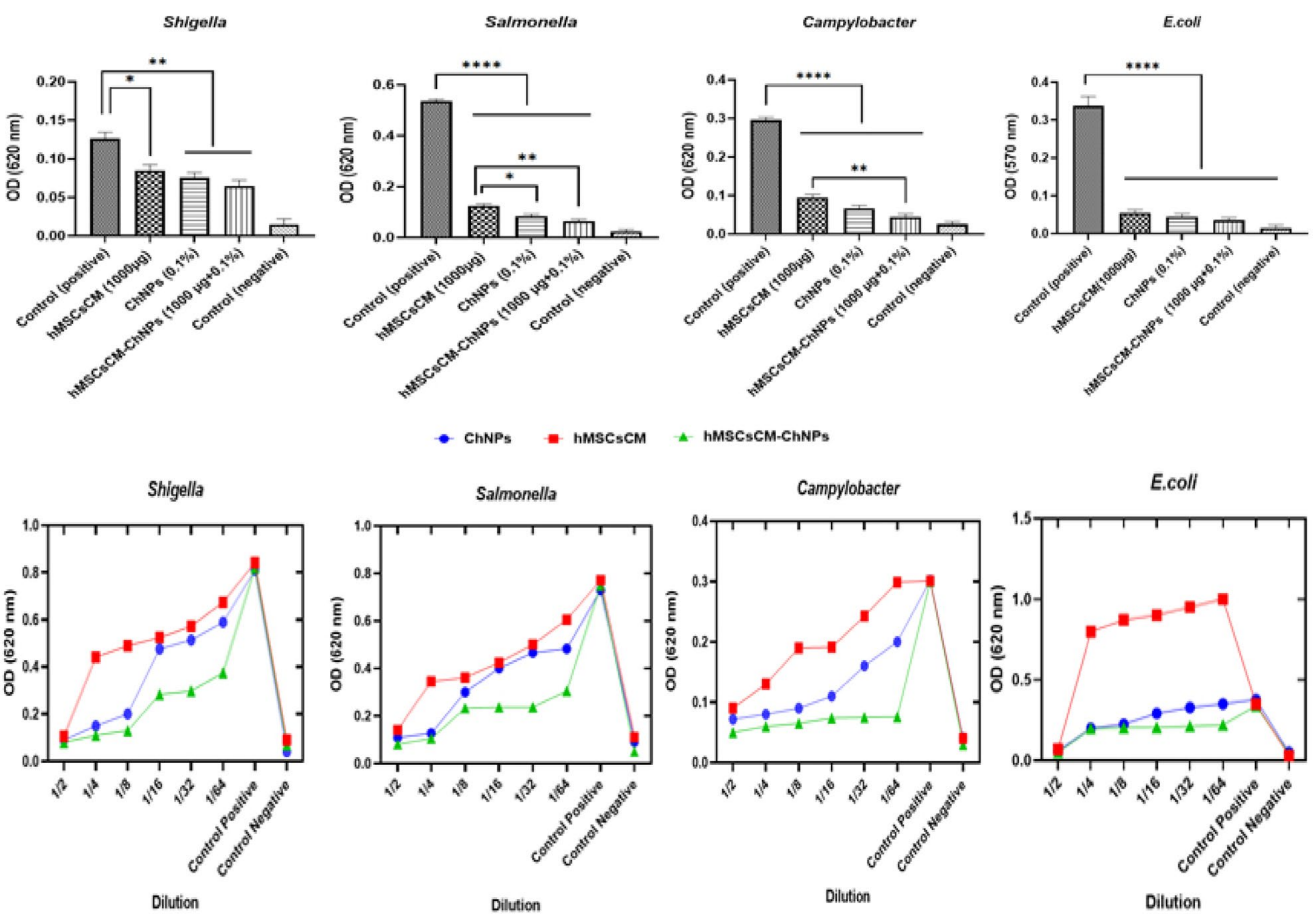
Inhibitory activities of the studied groups against the biofilm formation ability of common gastroenteritis bacteria. Bars represent the average results of triplicate experiments ± SD. The obtained results were analyzed by ANOVA test. *P* < 0.05: the mean difference was significant, *p-value < 0.05, **p-value < 0.01, ***p-value < 0.001, ****p-value < 0.0001), and *ns* no significant.

The effectiveness of the studied groups in diminishing the biofilm formation ability of common gastroenteritis bacteria was evaluated in-vitro. As shown in Fig. 5, the inhibitory activity of the studied groups against bacterial biofilm formation was photometrically measured at OD = 570 nm. The antibiofilm activity of the new construct (hMSCsCM-ChNPs: 1000 μg/ml and 0.1%) was higher compared to each of its components (ChNPs and hMSCsCM). The results showed that the new composite (hMSCsCM-ChNPs) exhibited a higher inhibitory activity against bacterial biofilm formation than hMSCsCM or ChNPs. The inhibitory activity of hMSCsCM-ChNPs compared to hMSCsCM was significant against *C. jejuni* ATCC29428 and *S. enterica* ATC13076. These findings highlight the synergistic effect of hMSCsCM and ChNPs. HMSCsCM-ChNPs at dilutions of 1:2 significantly promoted antibiofilm effects than those at a dilution of 1:64 (P < 0.05) against common gastroenteritis bacteria (*S. dysenteriae* PTCC1188*, S. enterica* ATCC13076, and *C. jejuni *ATCC29428) except for* E*. *coli* ATCC25922. Figure 5 shows the inhibitory activity of the studied groups against bacterial biofilm formation, which was photometrically measured at OD = 620 nm.

### In-vivo antibacterial activity in infected mice

In-vivo antibacterial activities of the new composite (hMSCsCM-ChNPs) and its components (ChNPs and hMSCsCM) against common gastroenteritis bacteria were evaluated in infected mice (Table 3). Fecal samples of mice were collected after treatment with hMSCsCM-ChNPs, ChNPs, and hMSCsCM and evaluated for the presence of the studied bacteria. The amount of bacteria decreased 48 and 72 h after treatment more in the group treated with hMSCsCM-ChNPs that in the groups treated with hMSCsCM and ChNPs. The results showed that the prepared composite (hMSCsCM-ChNPs) was more effective in treating intestinal bacterial infections than the other groups. None of the common gastroenteritis bacteria were detected in the negative control group.

**Table 3:** In-vivo antibacterial activities of the new composite (hMSCsCM-ChNPs) and its components (ChNPs and hMSCsCM) against common gastroenteritis bacteria evaluated in this study.

Pathogen	Minimum inhibitory concentration (24 h)	Minimum bacterial concentration (48 h)	Minimum bacterial concentration (72 h)
*Escherichia coli* ATCC 25922			
ChNPs	> 10^6^	10^5^	10^3^
hMSCsCM	> 10^6^	10^5^	10^2^
HMSCsCM-ChNPs	> 10^6^	80	60
*Campylobacter jejuni* ATCC 29428			
ChNPs	> 10^6^	10^5^	10^3^
hMSCsCM	> 10^6^	10^4^	10^3^
hMSCsCM- ChNPs	> 10^6^	300	250
*Salmonella enterica* ATCC 13076			
ChNPs	> 10^6^	10^4^	10^4^
hMSCsCM	> 10^6^	10^3^	10^2^
hMSCsCM- ChNPs	> 10^6^	170	100
*Shigella dysenteriae* PTCC 1188			
ChNPs	> 10^6^	10^3^	10^2^
hMSCsCM	> 10^6^	80	60
hMSCsCM- ChNPs	> 10^6^	15	10

## Discussion

Gastroenteritis refers to the inflammation and infection of the gastrointestinal tract mucus membranes. The disease could cause symptoms such as mild to severe diarrhea or vomiting and even death, particularly in children^1^. In recent years, the use of mesenchymal stromal cells (MSCs) has been considered as a new treatment strategy in the field of disease treatment due to their potential therapeutic properties. In the current research, MSCs supernatant and nanochitosan were employed as a new treatment method for common gastroenteritis bacteria. MSCs supernatant and nanochitosan showed robust antibiofilm and antibacterial activities against gastroenteritis bacteria.

In order to synthesize hMSCsCM-ChNPs composite, it was necessary to produce ChNPs. Positively charged nanoparticles were spontaneously formed by mixing chitosan and TPP as a crosslinking agent and forming compact complexes. Intramolecular and intermolecular crosslinks were formed in ChNPs by poly-anions. In ChNPs, a magnetic attraction was created between TPP with negative charge and chitosan amino groups with positive charge^19^.

Using SEM and DLS technologies, the features of ChNPs, including morphology, zeta potential, particle size, and polydispersity index (PDI), were confirmed. No severe agglomeration was detected in nanoparticles. Morphologically, ChNPs synthesized in this study were spherical in shape as previously reported^20^.

Recent studies have shown that MSCs express surface molecules. The cells examined in this study were negative for CD34 and CD45 but positive for CD44 and CD73 markers, these findings are in line with MSCs features ^21,22^. Controlled drug delivery and release technology has become a promising strategy for controlling diseases. In this study, hMSCsCM-ChNPs composite was constructed using mesenchymal stem cells and nanochitosan as a drug delivery system. After synthesis, ChNPs were subjected to characterization and loading with MSCs supernatant. The new construct (hMSCsCM-ChNPs) was confirmed by SEM technology (Fig. 2B). The morphological features of the new (hMSCsCM-loaded ChNPs) formulation were visible via SEM. The optimal amount of protein encapsulated within hMSCsCM-ChNPs was 75%. It was found that a high amount of protein was entrapped. The protein release pattern was examined in PBS medium at various pH values, Fig. 3 shows the obtained findings. The protein release rate decreased with decreasing pH. According to the results, the release rate of mesenchymal stem cells was higher at pH 7.4 compared to pH 1.2. This high release rate at pH 7.4 may be due to the chemical properties of MSCs and ChNPs, which are mainly affected by pH, resulting in diverse release patterns. In addition, the protein release rate increased with time, the protein release rate was higher after 72 h compared to the shorter time intervals examined, indicating the long-term release of MSCs. According to these results, the limited and controlled diffusion allows the long-term release of MSCs and ChNPs. This is an important feature that could be utilized to deliver specific intestinal antigens^23^. The release trial showed that the synergistic effect of ChNPs and hMSCsCM improved the protein release performance. Therefore, the results of this study suggest a new idea for the treatment of gastroenteritis bacteria and the release of proteins.

Chitosan, as a cationic antibacterial agent, and its derivatives have fascinating antibacterial activities. This substance has been extensively investigated for its antimicrobial activities against fungi as well as Gram-negative and Gram-positive pathogens. The mechanisms of antibacterial activity of chitosan are as follows. Positively charged chitosan binds to the amino group of bacterial cell surface components and disturbs cell membranes, resulting in microbial cell death, or coats the bacterial surface to inhibit the leakage of intracellular compounds. Another mechanism is through the inhibition of DNA/RNA and protein synthesis^5,24^.

Cell therapy using mesenchymal stem cells (MSCs), as a vital part of the innate immune system, is considered as a promising treatment option for diseases. In addition to neonatal tissues, these cells could be collected from different adult tissues, including peripheral blood, inner organs, bone marrow, and adipose tissue. Some studies have shown that MSCs could exhibit immunosuppressive or immune-enhancing properties^25^.

Several investigations have shown that the beneficial effects of MSCs could be due to the presence of soluble proteins, secreted vesicles, and antimicrobial peptides (AMPs), also known as host-defense peptides, including hepcidin, defensin-2, human cathelicidin (hCAP-18/LL-37), and β- lipocalin 2. Recently, clinical studies have shown that BM-isolated MSCs supernatant displays antibacterial effects against some bacteria due to the presence of antimicrobial peptides. MSCs supernatant could display bactericidal activity against Gram-negative and Gram-positive bacteria, including *E. coli*, *Pseudomonas aeruginosa*, and *S. aureus*^26^.

The current study results showed that the combination of ChNPs and hMSCsCM could be used as an efficient therapeutic strategy to inhibit the growth of bacteria. This combination may have a synergistic effect. But the mechanism of action of this synergy is not well understood. Several investigations have reported the antimicrobial effects of ChNPs^27,28^.

It is suggested that ChNPs in this construct probably interact with structures on the surface of Gram-negative (e.g., lipopolysaccharides and proteins) and Gram-positive bacteria (e.g., peptidoglycan and teichoic acids). As a result, changes in cell membrane permeability lead to the escape of intracellular components and ultimately cell death.

In addition, the bacterial cell wall is suggested to be more labile to the infiltration of proteins secreted by MSCs (including LL37), effectors, and antimicrobial peptides. Therefore, the combination of MSCs and chitosan could play a significant role in disrupting the cell wall, inhibiting mRNA and protein synthesis, and neutralizing LPS (lipopolysaccharides). Biofilm formation in pathogenic bacteria is a protection mechanism against microbicides and antibiotics. This mechanism prevents antimicrobials from entering the bacterial cell surface. Due to the presence of polymeric matrices in bacterial biofilms, antimicrobials could not easily access the bacterial cell surface components. The antibiofilm property of chitosan is attributed to the presence of amino groups of N-acetylglucosamine monomers. Negatively charged biofilm components include: e-DNA, extracellular proteins, and EPS (extracellular polymeric substances), extracellular proteins could interact with positively charged chitosan. As a result, chitosan could pass through the biofilm and destroy the bacteria^24^.

In line with this study results, several current studies have shown that chitosan could be used as a potential antibiofilm agent^29,30^. In a study, the anti-quorum sensing (anti-QS), antibiofilm, and antibacterial activities of canine BM-derived hMSCsCM were investigated in-vitro. The results showed that canine BM- derived hMSCsCM significantly inhibited bacterial growth and biofilm formation of *E. coli* and *S. aureus*^31^. In another study, it was demonstrated that antibacterial peptides of human umbilical cord MSCs (hUCMSCs) influenced *P. aeruginosa* biofilm formation through reducing the biosynthesis of gene-encoded polysaccharide proteins^32^.

In the present research, MSCs supernatant exhibited a dose-dependent inhibitory activity against bacterial biofilm formation, which may be due to modulation of quorum sensing, inhibition of peptidoglycan synthesis, and disruption of microbial membrane structures. Furthermore, the integration of MSCs and chitosan could increase the interaction between these components and microbial cell membranes and inhibit the growth of Gram-positive and Gram-negative pathogens. Previous studies have shown that MSCs and hMSCsCM-ChNPs could inhibit biofilm formation of *V. cholera*^8,33^. In this study, the effects of hMSCsCM and ChNPs on biofilm formation of common gastroenteritis bacteria were evaluated for the first time.

According to these results, the combined use of ChNPs and hMSCsCM could be regarded as an adjunct treatment for bacterial gastroenteritis. In this study, a new drug delivery system was proposed for the treatment of gastrointestinal infections. hMSCsCM-ChNPs was designed and evaluated as a new treatment strategy and exhibited potent antibiofilm and antimicrobial activities against common gastroenteritis bacteria, which may be mediated by the synergistic effect of hMSCsCM and ChNPs.

Therefore, the combination of ChNPs and hMSCsCM could be used as an antibiofilm and antibacterial drug delivery tool in the treatment of intestinal infections to deal with the resistance and colonization of gastroenteritis bacteria and as a reactive antimicrobial agent both individually and in combination with existing antimicrobials to enhance their antimicrobial effects. The new formulation and its components were characterized and evaluated for the first time against common gastroenteritis bacteria. There are few studies on the direct or indirect mechanisms of action of antimicrobials against intestinal bacteria to summarize and analyze the results. Therefore, further comprehensive investigations are needed to support the efficiency of mesenchymal stem cells for clinical applications.

## Conclusion

In conclusion, hMSCsCM-ChNPs exhibited highly efficient antibiofilm and antibacterial activities against bacteria. According to the preliminary findings, hMSCsCM-ChNPs in combination with existing antimicrobials could be used as another therapeutic method to effectively control fatal gastrointestinal bacterial infections, especially diarrhea as one of the main public health concerns. Furthermore, this new formulation showed a better release profile in simulated physiological conditions of intestinal lumen. Therefore, hMSCsCM-ChNPs might be valuable for numerous pharmaceutical applications.

## Data Availability

The datasets used and/or analyzed during the present research are accessible upon request from the corresponding author with no restriction.

## Abbreviations

WHO: World Health Organization
CFU: Colony forming units
SEM: Scanning electron microscopy
DLS: Dynamic light scattering
ChNPs: Chitosan nanoparticles
MSCs: Human mesenchymal stem cell
hMSCsCM: Human mesenchymal stem cell-derived conditioned medium
LB: Luria–Bertani
BHI: Brain-heart infusion
IHC: Immunohistochemistry
EE: Entrapment efficiency
SS agar: *Salmonella-shigella* agar
EMB: Eosin methylene blue
PBS: Phosphate-buffered saline
